# The intrinsic GTPase activity of the Gtr1 protein from *Saccharomyces cerevisiae*

**DOI:** 10.1186/1471-2091-13-11

**Published:** 2012-06-24

**Authors:** Palanivelu Sengottaiyan, Cornelia Spetea, Jens O Lagerstedt, Dieter Samyn, Michael Andersson, Lorena Ruiz-Pavón, Bengt L Persson

**Affiliations:** 1 School of Natural Sciences, Linnaeus University, SE-391 82, Kalmar, Sweden; 2 Department of Biological and Environmental Sciences, University of Gothenburg, SE-405 30, Gothenburg, Sweden; 3 Department of Experimental Medical Science, Lund University, SE-221 84, Lund, Sweden; 4 Laboratory of Molecular Cell Biology, Institute of Botany and Microbiology, Katholieke Universiteit Leuven, and Department of Molecular Microbiology, Flanders Institute of Biotechnology (VIB), Kasteelpark Arenberg 31, BE-3001, Leuven-Heverlee, Flanders, Belgium

**Keywords:** Gtr1, GTPase, Intrinsic tryptophan fluorescence, Rag GTPase, Cysteine mutagenesis, Switch region

## Abstract

**Background:**

The Gtr1 protein of *Saccharomyces cerevisiae* is a member of the RagA subfamily of the Ras-like small GTPase superfamily. Gtr1 has been implicated in various cellular processes. Particularly, the Switch regions in the GTPase domain of Gtr1 are essential for TORC1 activation and amino acid signaling. Therefore, knowledge about the biochemical activity of Gtr1 is required to understand its mode of action and regulation.

**Results:**

By employing tryptophan fluorescence analysis and radioactive GTPase assays, we demonstrate that Gtr1 can adopt two distinct GDP- and GTP-bound conformations, and that it hydrolyses GTP much slower than Ras proteins. Using cysteine mutagenesis of Arginine-37 and Valine-67, residues at the Switch I and II regions, respectively, we show altered GTPase activity and associated conformational changes as compared to the wild type protein and the cysteine-less mutant.

**Conclusions:**

The extremely low intrinsic GTPase activity of Gtr1 implies requirement for interaction with activating proteins to support its physiological function. These findings as well as the altered properties obtained by mutagenesis in the Switch regions provide insights into the function of Gtr1 and its homologues in yeast and mammals.

## Background

The small GTPases of the Ras superfamily have been implicated in diverse cellular functions, including regulation of ion channel activity, cytoskeleton reorganization, nucleocytoplasmic transport, vesicular trafficking, cell proliferation and differentiation [1]. All Ras-GTPases share conserved amino acid sequences, the G1 to G5 motifs, which regulate the GDP/GTP exchange and GTP hydrolysis, and thus trigger multiple intracellular signaling cascades [2]. Switch I and Switch II are flexible regions around G2 and G3, respectively, which change conformation upon GTP/GDP exchange, thus facilitating interactions with its effectors, GTPase-activating proteins (GAPs) and guanine nucleotide exchange factors (GEFs) [3,4]. Ras-GTPases generally exhibit slow dissociation of GDP for GTP and low intrinsic GTP hydrolysis rate, which are strictly enhanced by GEFs and GAPs, respectively [5].

The Gtr1 protein of *Saccharomyces cerevisiae* (*S. cerevisiae)* is a multifunctional GTP-binding protein, involved in phosphate acquisition through modulation of Pho84 transport activity [6], ribosome biogenesis [7] and epigenetic control of gene expression [8]. It has also been shown that the Gtr1 protein is a subunit of the EGO/GSE complex, which is indispensible for intracellular sorting of the amino acid permease Gap1 [9], Gtr1 has been implicated in regulation of the TOR signaling cascade in response to amino acids [10]. Most recently, it has been proposed that the Switch regions are essential for activation of the TOR1 complex (TORC1) [11]. Despite an otherwise low sequence similarity, the G motifs in Gtr1 display high conservation with other Ras GTPases, and are located within the N-terminal GTPase domain of the protein [12]. The C-terminal domain of the protein has been implicated in self-interaction [13] and protein-protein interactions [11]. In contrast to Ras homologues, the Gtr1 protein lacks lipid modification motifs in its C-terminal region, and the G4 motif (H/NKXD) contains a histidine instead of an asparagine residue [6].

Gtr1 belongs to the distantly related RagA family, which displays a low sequence similarity with the Ras, Rab, Ran, Arf and Rho proteins [7,13]. The Gtr1 protein displays 52 % and 47 % sequence identify with the mammalian Rag GTPases RagA and RagB, respectively [7]. Notably, recent studies have shown that the Gtr1 and RagA proteins share a similar mechanism of amino acid-mediated TOR activation, indicating that these proteins are functionally conserved in eukaryotes [14]. *S. cerevisiae* also contains Gtr2, which corresponds to RagC/RagD in humans. Like the mammalian Rag GTPases, GTP-bound Gtr1 and GDP-bound Gtr2 form a stable heterodimeric complex *in vitro*[13]. The GTP-bound form but not the GDP-bound Gtr1 interacts with itself, whereas Gtr2 can interact with itself only in the presence of GTP-bound Gtr1 [13]. Most recently, the 3D-structure of the Gtr1-Gtr2 complex in the GMP-PNP bound form was resolved at 2.8 Å resolution [11]. The structure has brought insights into the location of G domains and Switch regions. Based on this, it was proposed that upon nucleotide exchange the Switch regions change conformation, allowing for interaction with Raptor and activation of TORC1 [11]. In the same report, it was shown that the C-terminal domains of the two proteins mediate heterodimeric complex formation that is indispensible for activation of TOR signaling pathways.

Previous studies have localized the Gtr1 protein both to the nucleus and to the cytoplasm [15], where it interacts in a GTP-dependent manner with diverse cytoplasmic and nuclear proteins, such as Ego1, Yrb2, Nop8 and Ego3 [16-19]. Based on competition studies, it was proposed that the Gtr1 protein displays higher affinity for GTP than for GDP [13]. The GTP-bound form of Gtr1 interacted with the Rpc-19 subunit of RNA polymerases I and III in yeast two-hybrid assays, indicating a role in the assembly of RNA polymerase [20]. Recently, the Vam6 protein, known as a GEF for Ypt7, was shown to assist the exchange of GDP/GTP on the Gtr1 protein *in vitro*[14]. Moreover, Binda *et al*. [21] have reported that Vam6 activates the Gtr1 subunit of the EGO complex during TORC1 function.

Gtr1 harbors all the necessary structural elements for functioning as a GTPase. Previous attempts to estimate the GTPase activity of Gtr1 [13], RagA [7,] and RagC proteins [16] resulted in either none or too low activity to be measured. Here we quantify the intrinsic GTP hydrolytic activity of purified recombinant Gtr1 protein and several variants using a radioactive GTPase assay, and in addition study their tryptophan fluorescence emission properties.

## Methods

### Gene cloning, heterologous expression and purification

*S. cerevisiae* full-length Gtr1 proteins (wild-type, cysteine-less (C-less), Arg37Cys and Val67Cys) were expressed in *Escherichia coli* and purified in a single step by affinity chromatography essentially as described [12]. The complete procedure is provided in Additional file 1. The protein expression levels and purity of the preparations were assayed as described [12]. The oligomeric state of Gtr1 was assayed by native gel electrophoresis as described [22]

### Assay of GTPase activity

The GTPase assay was carried out using purified recombinant protein at a final concentration of 0.15 mg ml^-1^ (4 μM), 185 nM GTP and 15 nM [α-^32^P] GTP (3000 Ci/mmol, 1 Ci = 3.7x10^10^ Bq; PerkinElmer, Boston, USA) in a 50 μl containing assay buffer (25 mM HEPES/NaOH, pH 7.4, 5 mM KCl, 5 mM MgCl_2_ and 100 mM sucrose) as described in Ref. [23].

The enzymatic reaction was carried out at 37 °C for the indicated time periods (0, 15, 30, 60 and 120 min), and terminated by the addition of an equal volume of 4 M formic acid. A volume of 2.5 μl (62.5 nCi) quenched reaction mixture was spotted onto a poly (ethyleneimine)-cellulose plate (Merck, Germany) and the nucleotides were separated by thin-layer chromatography using 0.75 M KH_2_PO_4_ (pH 3.65) as elution buffer. The radioactive nucleotides were detected by phosphorimaging (BAS-1500, Fuji, Japan). The radioactive GDP spots were quantified using MultiGauge software (Fuji, Japan). The data are given as means ± SD and were obtained from 2–3 independent experiments. Initial velocities for GTP hydrolysis were measured using the same assay conditions at varying [α-^32^P] GTP concentrations (0.1 to 1.2 μM) for 60 min. The data were plotted as a function of GTP concentration, and fitted to the Michaelis-Menten equation. A Lineweaver-Burk plot of the data was used to determine the values for the Michaelis-Menten constant (K_m_) and the maximum velocity (V_max_) parameters. The catalytic constant k_cat_ was calculated as the V_max_/E_t_ ratio, where E_t_ is the enzyme concentration.

### Intrinsic tryptophan fluorescence

Tryptophan (Trp) fluorescence spectra of purified recombinant Gtr1 proteins were recorded using a Fluoromax-3 spectrofluorometer (Horiba Jobin Yvon, Japan) in the presence of guanine nucleotides. The interaction of the protein with guanosine 5’-*O*-(3-thiotriphosphate) (GTPγS) or GDP was measured using 0.019 mg ml^-1^ (0.50 μM) protein and 25 μM nucleotides in the GTPase assay buffer. The reaction mixture was incubated for 120 min at 37 °C prior to fluorescence measurements [24]. The intrinsic protein fluorescence was excited at 297 nm, and emission spectra were recorded in the range 297 to 400 nm at 25 °C. The spectra of free nucleotides in assay buffer were subtracted from the collected sample dataset.

### Sequence alignment

**Figure 1:**
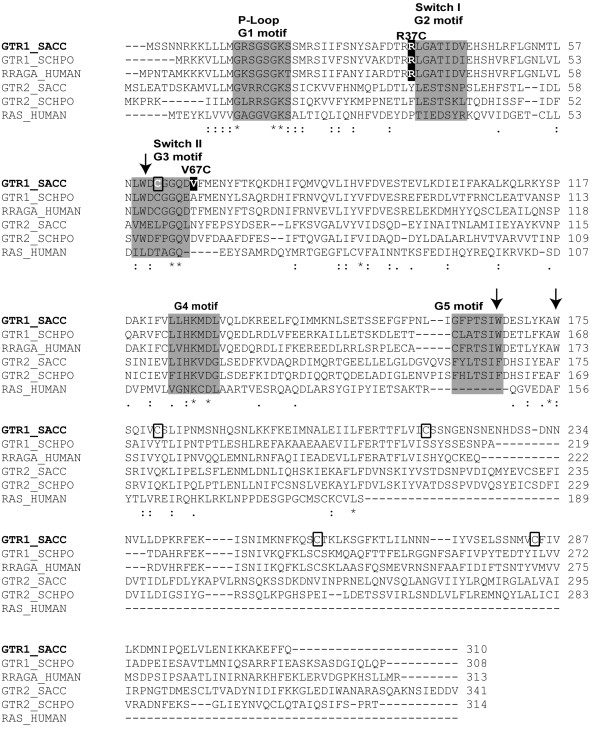
**Location of G motifs in the primary structure of the Gtr1 protein from*S. cerevisiae*.** Multiple sequence alignment of GTR1_SACC with its closest homologues GTR2_SACC, GTR1_SCHPO and Gtr2_SCHPO, RRAGA_HUMAN and HRAS_HUMAN was constructed using ClustalW program, in which SACC, SCHPO and HUMAN represent *Saccharomyces cerevisiae*, *Schizosaccharomyces pombe and Homo sapiens,* respectively. The G1 to G5 sequence motifs in the GTP binding domain of Gtr1 protein are depicted in the gray shaded box. The star (_*_), single (·) and double (**:**) dots represent identical, conserved and semi-conserved residues in the respective regions. The positions of cysteine and tryptophan residues are indicated by rectangular boxes and arrows, respectively. The residues Arg37 and Val67 mutated to cysteines in this work are highlighted on a dark background.

**Figure 2:**
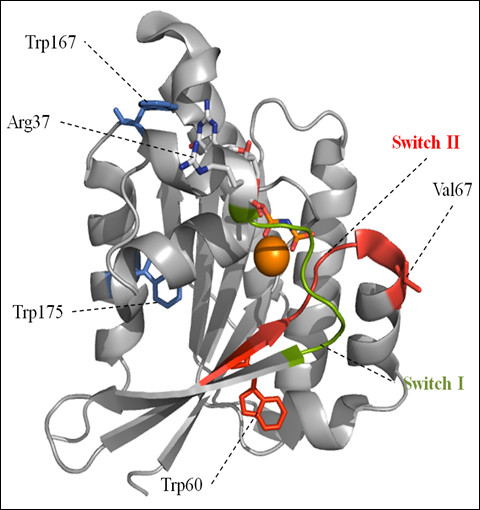
**Schematic representation of nucleotide binding domain of Gtr1 with bound GMPPNP, derived from the X-ray crystal structure of the Gtr1-Gtr2 complex (PDB id: 3R7W).** Switch I and II regions are shown in green and red, respectively. The side chains of Arg37 and Val67 are shown in blue and red, respectively. The positions of tryptophan residues are shown as Trp60 (red), and Trp167 and 175 (blue). Bound Mg^2+^ is shown in orange. The molecular graphic image was generated using PyMOL.

Sequence alignments of Gtr1 from *S. cerevisiae* with other yeast and mammalian members of RagA family and with human H-Ras were obtained using ClustalW (Figure1). The location of G motifs, Switch regions and residues mutated in this work are highlighted in the alignment. The position of the nucleotide-binding domain of Gtr1 (residues 1–185), of the Switch I and II regions, of the mutated Arg37 and Val67 residues, and the tryptophan residues, Trp60, Trp167 and Trp175, are indicated in the X-ray crystal structure of Gtr1 (Figure2).

## Results and discussion

**Figure 3:**
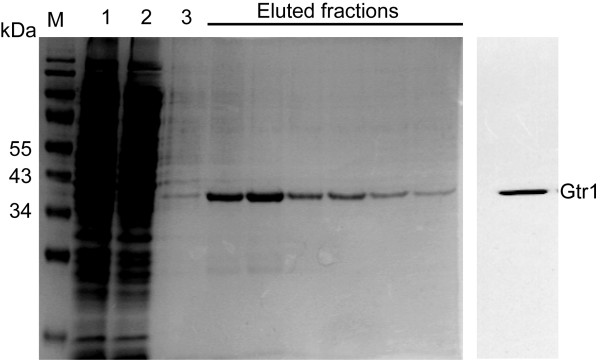
**Purification of recombinant His**_**6**_**-Gtr1 wild-type protein by immobilized metal affinity chromatography.** Coomassie Brilliant Blue stained 12 % (w/v) acrylamide SDS gel containing following samples: M, prestained protein molecular weight marker (kDa); lane 1, aliquot of sonicated total cell lysate; lane 2, aliquot of soluble fraction of total cell lysate after centrifugation at 40,000 g; lane 3, flow-through protein from the Ni^2+^-NTA column, and lanes 4–9, eluted fractions of His_6_-Gtr1 wild-type protein. Prior to the GTPase assay, an aliquot of eluted His_6_- Gtr1 wild-type protein from the Ni^2+^-NTA column was verified by Western blot analysis (right panel) using anti-His antibody.

The GDP/GTP exchange and intrinsic GTP hydrolytic activity are the most important functions for all regulatory GTPases. In order to characterize the properties of Gtr1, an N-terminal His_6_-tagged version of the full length Gtr1 protein was stably over-expressed using a bacterial production system and purified by a single step affinity chromatography [12]. Based on SDS-PAGE analysis, the purity of the recombinant protein was estimated to 95 % and the M_r_ to 36 kDa. The identity was confirmed by Western blot analysis using an anti-His antibody (Figure3). The oligomeric state of the recombinant protein is monomeric since the recombinant protein migrates at 36 kDa in native gel electrophoresis (Additional file 2: Figure S1).

### Intrinsic tryptophan fluorescence of the purified Gtr1 protein

**Figure 4:**
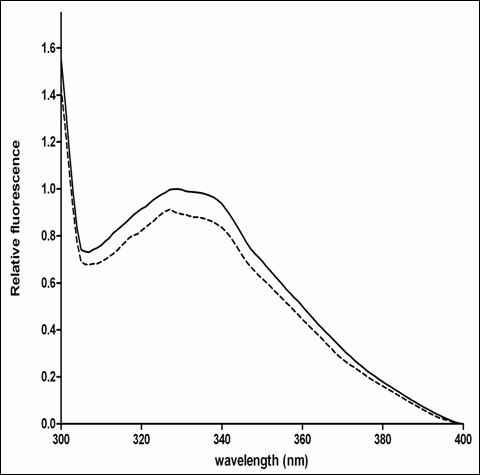
**Representative relative tryptophan fluorescence spectra of purified Gtr1 wild-type protein in the GDP (full line) and GTP**γ**S (hatched line) states.** Data are representative of two replica experiments. The spectra of each sample were obtained after normalization to the fluorescence level of the Gtr1-GDP complex.

Trp fluorescence has been widely used as a tool to study structural changes upon nucleotide binding to Ras GTPases [23,24] The Gtr1 protein harbors three Trp residues, namely Trp60, Trp167 and Trp175 (Figures 1 and 2). Conformational alterations upon nucleotide binding to Gtr1 could result in changes in the local environment of these Trp residues in the protein structure. Figure4 shows the fluorescence emission spectra of purified Gtr1 wild-type protein in the presence of GDP or GTPγS. When loading the Gtr1 wild-type protein with nucleotides, we observed a blue shift of 2 nm in the tryptophan fluorescence emission between the GTPγS-bound form (329 nm) and the GDP-bound form (327 nm) (Table1). This indicates a more hydrophobic localization of the tryptophan residues upon GDP binding, and thus a more compact overall conformation. Furthermore, the data presented in Figure4 indicate that the recombinant Gtr1 wild-type protein has retained the nucleotide binding functionality. Taken together, our results indicate that Gtr1, like other GTPases, can adopt two distinct conformations, corresponding to the GTP-bound and GDP-bound state, which can be monitored by tryptophan fluorescence. This tool has been used to study several mutants in the work, as described below. 

**Table 1:** The fluorescence emission intensity and λmax of Gtr1 and the mutations were measured as described in Methods

**Mutation**	**Relative intensity (GTPγS / GDP)**	**Emission spectra**	
		**λ**_**max**_**(GDP)**	**λ**_**max**_** (GTPγS)**
WT	0.91 ± 0.01	329 ± 1	327 ± 1
Cys-less	0.95 ± 0.02	327 ± 1	326 ± 1
Arg37Cys	0.90 ± 0.01	325 ± 1	324 ± 1
Val67Cys	0.96 ± 0.01	321 ± 2	316 ± 2

The peak intensity measurements in the GTPγS-bound form of each protein were normalized to those measured for the GDP-bound form. Wavelengths of the emission maxima measured are also presented for the GDP- and GTPγS-bound proteins. Data shown in the table are means±SD of two independent measurements.

### Intrinsic GTPase activity of the purified Gtr1 protein

**Figure 5:**
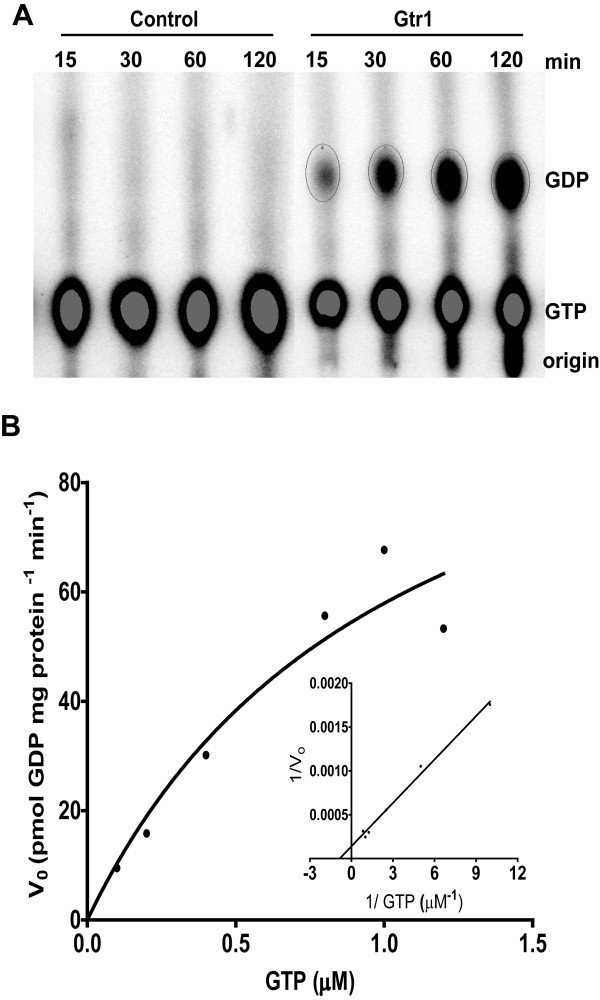
**The assay of GTPase activity for the purified Gtr1 wild-type protein using [**α**-**^**32**^**P]GTP. (A) – The GTPase activity was assayed as described in Methods.** As a control, the assay was also performed in the absence of protein. The nucleotides were separated by thin layer chromatography, and the radioactive spots visualized by phosphorimaging. **(B) - Michaelis Menten titration and Lineweaver-Burk plot (insert) of the GTPase activity.** Recombinant Gtr1 protein was incubated for 60 min at 37 °C with different concentrations of [α-^32^P] GTP. The initial rates were plotted versus GTP concentration. K_m_ and V_max_ were calculated from the Lineweaver-Burk plot. The correlation coefficient was 0.9933.

Previous attempts to measure the GTPase activity of Gtr1 and RagA proteins have failed [7,13], and thus the proteins were considered to lack GTPase activity in the absence of activators. A weak GTPase activity has been indicated for RagC [16]. Here we measure the intrinsic GTPase activity of Gtr1 using [α-^32^P] GTP and purified wild-type Gtr1 in assay conditions similar to those previously described [23]. As shown in Figure5A, as result of the hydrolytic activity, GDP was produced in amounts increasing with the incubation time in samples containing purified protein, whereas GDP production was absent in control samples devoid of protein. The chromatography plates were overloaded with radioactive sample, and therefore, the corresponding decrease in the GTP amount resulting from its hydrolysis could not be detected. The ratio between the amount of GDP and the total amount of GDP + GTP reached a maximum of 30 % at the incubation time of 120 min, in line with the previous report for RagC 16].

Next, we determined the enzymatic kinetic parameters of the protein under similar assay conditions as described in Methods. Values of 1.0 μM and 0.1 nmol GDP mg protein^-1^ min^-1^ were obtained for the K_m_ and V_max_ parameters by Michaelis-Menten analyses (Figure5B and insert). The obtained values were used to calculate the k_cat_ of the protein, 0.004 min^-1^. For reference, the k_cat_ values determined for three representative GTPases, namely G_ial_, Ras and EF-TU are 3, 0.3 and 0.003 min^-1^, respectively [25]. The finding of an extremely low GTPase activity implies a strict regulation, for example interaction with other proteins, such as Gtr2. In addition, Vam6 has been proposed as a GEF for Gtr1 [14,21]. It will be important to understand the way this key GTPase is regulated by activating proteins to control various processes.

### Role of Arg37 and Val67 in the GTPase activity of the Gtr1 protein

**Figure 6:**
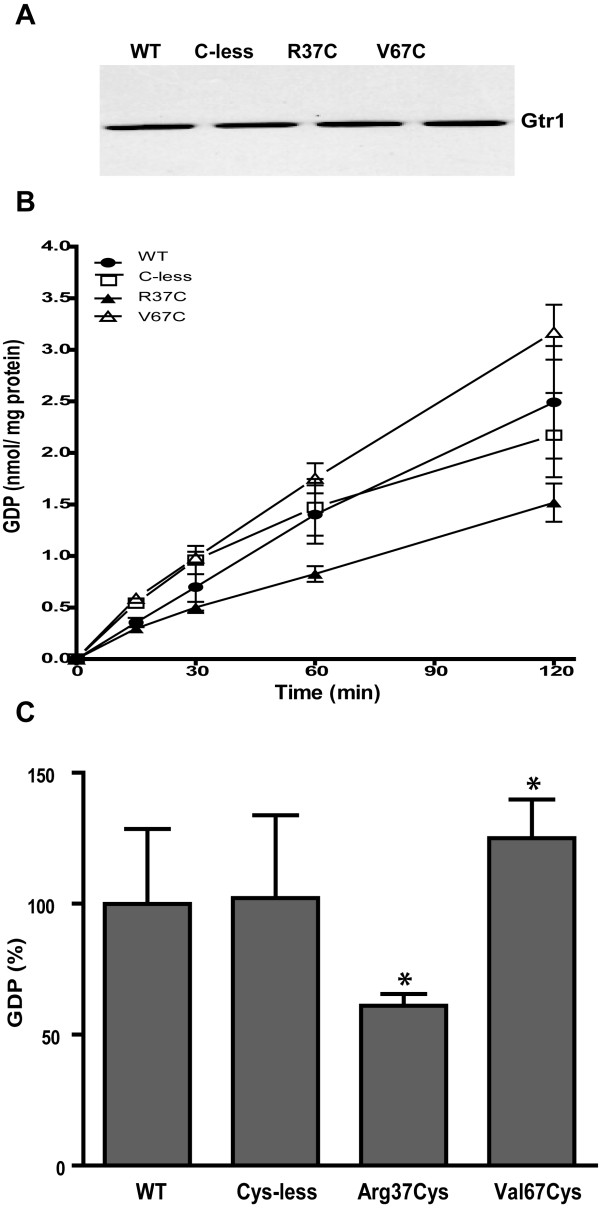
**Expression and activity assays of Gtr1 wild-type (WT), cysteine-less (Cys-less), Arg37Cys and Val67Cys proteins. (A)** Western blot analysis using anti-His antibody. **(B)** GTP hydrolytic activity of WT (·) Cys-less (❑), Arg37Cys (▴) and Val67Cys (Δ) shown as the amount of GDP produced as a function of time. **(C)** GTPase activity of Gtr1 mutants at 60 min of incubation relative to that for the WT protein. 100 % is equivalent to 1.76 nmol GDP mg^-1^ protein. The presented data are means ± SD (n = 2-3 independent experiments). *, Significantly different from WT (Student’s *t*-test P < 0.05).

We have previously used EPR spectroscopy analysis on site-directed mutants of Gtr1 and showed that only the N-terminal part of the protein harbors the structural elements involved in GTP binding [12]. In the same study, we examined the conformational changes upon GTP binding of Gtr1 in the regions around residues Arg37 and Val67. These residues are located at the border of Switch I and II region, respectively (Figures 1 and 2). Sequence analysis indicated that the Arg37 residue is highly conserved within the RagA family, but is not present in other Ras GTPases (Figure1). On the other hand, the Val67 residue is not conserved in either Rag or other Ras proteins. Residues Arg37 and Val67 were, however, chosen for mutagenesis since the activities of the two Switch regions could be monitored without completely abolishing GTP/GDP-binding. We have tested the functional importance of these residues in Gtr1 by measuring the GTPase activity of Arg37Cys and Val67Cys mutants constructed in a Cys-less background (Cys replaced by Ser). In addition to offer the ability to modify the cysteine thiol-group with spectroscopic probes, the relatively low bulkiness of the cysteine side chain as well as its chemical ambivalence makes the cysteine residue a suitable replacement to study the dynamics of the Switch regions. As shown in Figure1, the amino acid sequence of the Gtr1 protein contains five native cysteine residues, of which Cys62 and 180 reside in the N-terminal GTPase domain, whereas Cys217, 257 and 284 are located in the C-terminal domain. The creation of a functional Gtr1 Cys-less, in which serine residues replaced all five native cysteine residues, has been previously described [12]. All three mutant proteins (Cys-less, Arg37Cys and Val67Cys) were purified under similar conditions as the wild type (WT) Gtr1 protein. Western blot analysis shows similar levels of the expressed proteins (Figure6A). We first analyzed the GTPase activity of the Cys-less mutant. The amount of GDP produced by the Cys-less mutant was found to be slightly higher than that in the WT at 30 min, followed by a gradual decrease to WT levels during prolonged incubation with [α-^32^P]GTP (Figure6B). This result indicates that cysteine residues may not be essential for the GTPase activity, which is consistent with previous reports on other Ras GTPases [26,27]. The Arg37Cys mutant exhibits a diminished GTP hydrolytic activity (60 % of WT levels) throughout the incubation period (Figure6B and C). In contrast, the Val67Cys mutant displayed about 25 % higher GTPase activity than the WT protein. These results indicate the importance of Arg37 and Val67 for the intrinsic GTPase activity of Gtr1, in the absence of interacting proteins such as GEF and Gtr2.

### Intrinsic tryptophan fluorescence of the Gtr1 mutants

**Figure 7:**
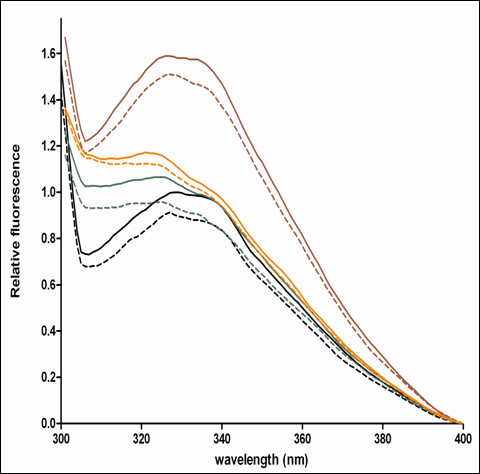
**Representative relative tryptophan fluorescence spectra of purified Gtr1 wild-type (black), Cys-less (red), Arg37Cys (green) and Val67Cys (orange) proteins in the GDP (full line) and GTP**γ**S (hatched line) states.** Data are representative of two replica experiments.

Next, we investigated if the effect of mutations on the GTPase activity could be explained by the dynamics in the protein structure. For this purpose, all mutant Gtr1 proteins were analyzed for tryptophan fluorescence in a similar manner as performed for the WT protein. As observed in the WT, the emission maxima in all mutant proteins, displayed a blue shift between GDP and GTPγS forms, (Figure7 and Table1), indicating that environmental changes for the Trp residues contribute to the observed blue shift. The blue shift from the GDP to the GTPγS states in Arg37Cys was the smallest (1 nm) whereas in Val67C is the largest (5 nm). Notably, a blue shift was also visible in both the GDP and GTPγS-bound states of all mutants relative to the WT, indicating a more hydrophilic environment for the Trp residues in the mutants. Again, Arg37Cys and Val67Cys distinguished themselves from the Cys-less mutant since they display the largest blue shifts relative to the WT in both nucleotide bound states. A third interesting difference was the higher fluorescence intensity observed for both the GTPγS and GDP forms in the Cys-less mutant as compared to all other samples, and might be explained by changes in the degree to which Trp fluorescence is quenched by neighboring residues in this mutant. The observed difference cannot be attributed to possible nucleotide-induced aggregation of the protein as similar levels of the protein were indicated in all samples by native PAGE analysis (Additional file 2: Figure S1). Moreover, this difference may have no relevance for the GTPase activity in the Cys-less mutant, which displayed WT levels. Taken together, the fluorescence data indicate that distinct conformational changes occur in the Arg37Cys and Val67Cys mutants that could impact on their performance during the GTPase assays (Figure6).

### Role of Arg37 in the crystal structure of Gtr1-Gtr2 complex

Recently, the crystal structure of the Gtr1-Gtr2 complex, with the two proteins bound to GMPPNP, was solved [11]. This structural model served as a template to design and analyze the role of RagA in activating TORC1. Mutational analysis of several residues indicated that the surface charge potential is of great importance for RagA to interact with Raptor, and thus being able to activate TORC1 [11]. Of the two adjacent arginines close to the Switch I region and which are conserved amongst the Gtr1 and RagA region (Arg36, Arg37), we have mutated Arg37 to a cysteine and have observed a change in hydrolytic activity. The removal of one of the two Arg residues might alter the surface charge to a degree where a minimal conformational alteration could influence the nucleotide interaction. Whether this charge alteration effectively results in a conformational change that impairs nucleotide binding is currently not known. Our results indicate that a replacement of Arg37 with a cysteine leads to a decreased hydrolytic activity, supporting the importance of this residue.

## Conclusions

Here we have employed a combination of structural, cysteine mutagenesis, radioactive GTPase assays and intrinsic tryptophan fluorescence approaches to study the biochemical activity of Gtr1 from *S. cerevisiae.* The data obtained reveal a very low intrinsic GTPase activity of the Gtr1 protein as compared to Ras GTPases, implying requirement for activating proteins, as previously reported [14,21]. This activity was found altered in Arg37Cys and also Val67Cys mutants of the Switch regions and associated with conformational changes, which are distinct from those in the WT and cysteine-less mutant. Despite the fact that both mutated residues are not fully conserved amongst Gtr1 homologues, alterations made in those positions have an influence on the GTPase activity and fluorescence. This indicates that both residues are of importance for the functionality of Gtr1. Our findings provide insights into the function of Gtr1 and its homologues in yeast and mammals.

## Abbreviations

GAP: GTPase-activating protein; GEF: guanine nucleotide exchange factor; GTPγS: guanosine 5’-*O*-(3-thiotriphosphate); TORC1: TOR complex 1; Trp: tryptophan; WT: wild type.

## Authors’ contributions

PS participated in experimental design and conducted the bulk of experimental work from protein overexpression to data analysis, and article writing. CS participated in the design and analysis of the GTPase activity data and in writing of the article. JOL generated the cysteine mutants and participated in the discussion of the fluorescence data. DS produced the structural figure and analyzed the fluorescence data. LRP participated in data processing and figure preparations. MA participated in protein expression and purification. BLP participated in the design, coordinated the experiments and writing of the article. All authors have read and approved of the final manuscript.

## Supplementary Material

Additional file 1 **Supplemental methods - Expression in *Escherichia coli*and purification of Gtr1 protein **[12]**.** Click here for file

Additional file 2 **Figure S1 - Assessment of the oligomeric state of recombinant Gtr1 protein by native gel electrophoresis.** Figure S2 - Stability of the nucleotide-bound Gtr1 Cys-less protein assayed by native gel electrophoresis and Western blotting. Click here for file
