# Supplementary material for: The intrinsic GTPase activity of the Gtr1 protein from Saccharomyces cerevisiae
Source: BMC Biochem. 2012 Jun 24;13:11. doi: 10.1186/1471-2091-13-11 (PMC3477016; doi:10.1186/1471-2091-13-11)
Supplement: Additional file 1 — Supplemental methods - Expression in Escherichia coliand purification of Gtr1 protein [12]. [file 1471-2091-13-11-S1.doc]

**Additional file 1**

**Supplemental Methods**

**Expression in *E*. *coli* and purification of Gtr1 protein**

The pTrcHisB (Invitrogen) was used to express the His_6_-Xpress-GTR1 fusion protein and the cysteine-less (Cys-less), Arg37Cys and Val67Cys variants as previously described [1], with the modification that the *EcoRI* and *NotI* were used as restriction sites. All constructs were cloned into pET28a(+) plasmid, and verified by DNA sequencing. The plasmid pET28a(+)/*GTR1* constructs were transformed into *E. coli* BL21 (DE3) cells and grown in LB medium containing kanamycin (100 μg/ml) at 37 ºC with continuous shaking until an A_600_ of 0.6 was reached. The cells were induced in LB with 1 mM IPTG at 25ºC for 18 h, and collected by centrifugation (3,000 g, 8 min). The pellets were washed with ice-cold 10 mM Tris/HCl buffer (pH 8.5) containing 100 mM NaCl and 1 mM EDTA, followed by centrifugation. The final pellet was resuspended in ice-cold buffer A (50 mM Tris/HCl, pH 7.4, 200 mM NaCl, 10 mM MgCl_2_, 10% (w/v) glycerol, 0.5% (v/v) Triton X-100, 30 mM imidazole, 5 mM β-mercaptoethanol, 100 μM Tris (2-carboxyethyl) phosphine and 1 mM PMSF). The suspension was incubated with lysozyme (Sigma, USA), 1 mM PMSF and protease inhibitor cocktail (Sigma, USA) for 40 min on ice, followed by sonication. The incubation was continued for additional 30 min followed by sonication. The lysate was centrifuged (40,000 g, 30 min) at 4 ºC. The supernatant was filtered using a 0.45 μm filter and loaded onto a HisTrap nickel affinity column (GE Healthcare, USA). The unbound protein was removed with buffer A and then the bound His-tagged Gtr1 protein was eluted by a linear gradient with Buffer B (Buffer A with 1000 mM imidazole). The larger proportion of the bound protein was eluted in the range of 400-500 mM imidazole concentration.

**References**

1. Lagerstedt JO, Reeve I, Voss JC, Persson BL: **Structure and function of the GTP binding protein Gtr1 and its role in phosphate transport in *Saccharomyces cerevisiae*.** *Biochemistry* 2005, **44(2):**511-517**.**
