# Supplementary material for: The intrinsic GTPase activity of the Gtr1 protein from Saccharomyces cerevisiae
Source: BMC Biochem. 2012 Jun 24;13:11. doi: 10.1186/1471-2091-13-11 (PMC3477016; doi:10.1186/1471-2091-13-11)
Supplement: Additional file 2 — Figure S1 - Assessment of the oligomeric state of recombinant Gtr1 protein by native gel electrophoresis. Figure S2 - Stability of the nucleotide-bound Gtr1 Cys-less protein assayed by native gel electrophoresis and Western blotting. [file 1471-2091-13-11-S2.doc]

**Additional File 2**


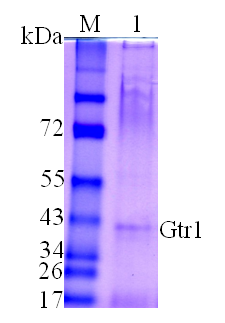


**Figure S1 - Assessment of the monomeric form of recombinant Gtr1 protein by native gel electrophoresis.** 10 µl of (2.5 µg of total protein) purified recombinant Gtr1 protein was mixed with the equal volume of native loading buffer (120 mM Tris-HCl (pH 6.8), 20% glycerol and 0.004 mg bromophenol blue). The above sample (lane 1) was resolved by 12% native PAGE and stained with Coomassie blue. The molecular masses of standard proteins are given on the left kDa.

**
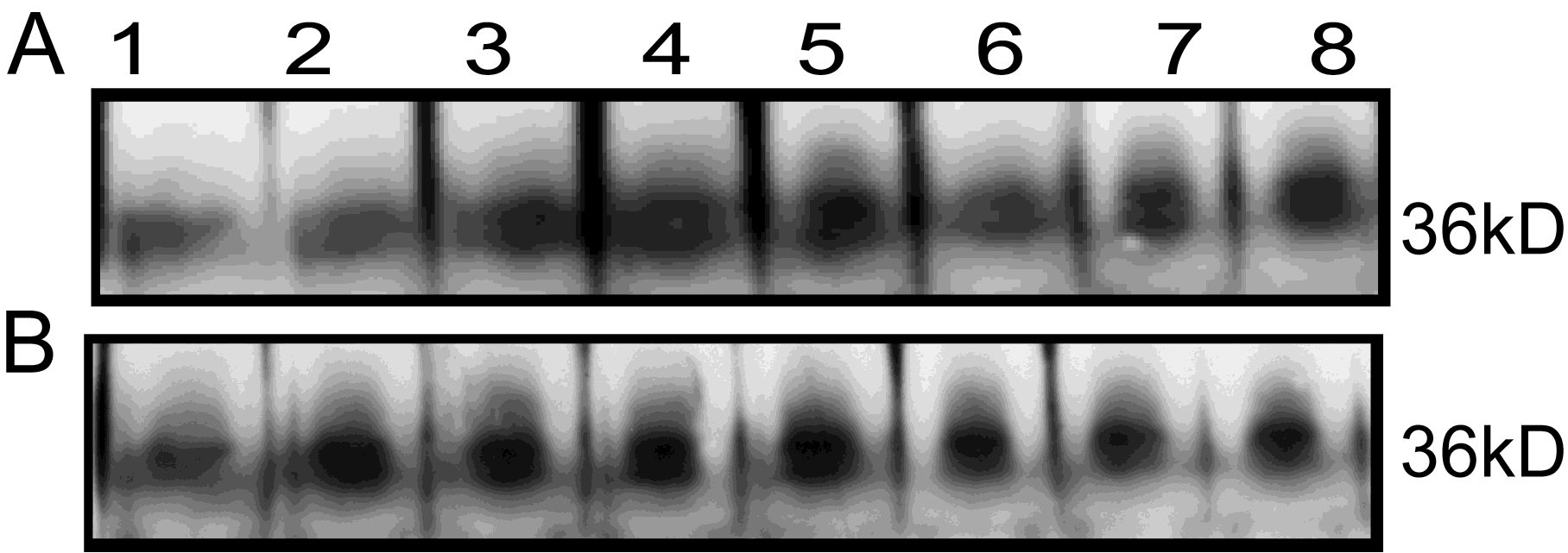
**

**Figure S2 - Stability of the nucleotide-bound Gtr1 Cys-less protein assayed by native PAGE and Western blotting.** 2.5 µg of purified Gtr1 wild-type or Cys-less protein were incubated for up to 2 h in the assay buffer in the absence or presence of 25 µM of nucleotides (GTPγS or GDP) at 37^o^C. **(A)** Lane 1, Cys-less incubated for 120 min; Lane 2, 3, 4, 5, 6, Cys-less incubated in the presence of GTPγS for 0, 15, 30, 60 and 120 min, respectively; lane 7, wild-type Gtr1 incubated for 120 min in the absence of GTPγS; lane 8, wild-type Gtr1 incubated for 120 min in the presence of GTPγS. **(B)** Lane 1, Cys-less incubated for 120 min; Lane 2, 3, 4, 5, 6, Cys-less incubated in the presence of GDP for 0, 15, 30, 60 and 120 min, respectively; lane 7, wild-type Gtr1 incubated for 120 min in the absence of GDP; lane 8, wild-type Gtr1 incubated for 120 min in the presence of GDP. Immunodetection was accomplished by use of anti-His antibody.
